# Supplementary material for: The Effectiveness of Interventions Delivered Using Digital Food Environments to Encourage Healthy Food Choices: A Systematic Review and Meta-Analysis
Source: Nutrients. 2021 Jun 30;13(7):2255. doi: 10.3390/nu13072255 (PMC8308236; doi:10.3390/nu13072255)
Supplement: Supplementary file 1 [file nutrients-13-02255-s001.zip › Nutrients Supp Files 29.06/Additional file 1 medline search strategy.pdf]

Additional file 1: Table S1: Medline search strategy.

Database(s): **Ovid MEDLINE(R) Epub Ahead of Print, In-Process & Other Non-Indexed Citations, Ovid MEDLINE(R) Daily and Ovid MEDLINE(R)** 1946 to September 29, 2020
Search Strategy:

#	Searches
1	(online or on-line).mp.
2	Online Systems/
3	Mobile applications/
4	((Phone or mobile) adj app*).ti,ab,kw.
5	Internet/
6	(website or web page or web-based).mp.
7	(cyber or virtual or digital or technolog* or software).mp.
8	((mobile or cellular or cell or smart) adj (phone* or telephone*)).ti,ab.kw.
9	(e-health or eHealth or electronic health).ti,ab.kw.
10	Computers/
11	User computer interface/
12	1 or 2 or 3 or 4 or 5 or 6 or 7 or 8 or 9 or 10 or 11
13	Nutrition*.mp.
14	(health* adj2 eat*).mp.
15	Fruit/ or fruit*.mp.
16	Vegetables/ or vegetable*.mp.
17	(canteen* or menu or cafeteria* or kiosk).mp.
18	Food service/
19	(calorie* or kiojoule*).mp.
20	Energy intake/
21	Energy density.mp.
22	Eating/
23	Feeding behavior/ or feeding behaviour.mp.
24	Nutrition* intake.mp.
25	Food habits/ or food/
26	Carbonated beverages/ or (soft drink* or soda or sweetened drink*).mp.
27	Dietary fats/
28	Confectionary.mp.
29	(school adj2 (lunch* or meal* dinner*)).mp.
30	(nutrition* adj2 program*).mp.

31	Exp diet/
32	Diet*.ti,ab.
33	Snacks/ or lunch/ or meals/ or breakfast/ or dinner.mp.
34	13 or 14 or 15 or 16 or 17 or 18 or 19 or 20 21 or 22 or 23 or 24 or 25 or 26 or 27 or 28 or 29 or 30 or 31 or 32 or 33
35	(purcahs* or buy or buying or bought or sale*).mp.
36	(order* or select*).ti,ab.
37	((consumer? or shopping) adj2 (choice? or behavio*)).mp.
38	(product? Adj3 (select* or choice*)).mp.
39	((grocer* or food* or fruit? Or vegetable? Or dairy or fish or fat? Or salt? Or sugar? Or sweet* or calori* or canteen or menu or soda or drink or beverage or meal or cafeteria or snack or lunch or breakfast or diet*) adj2 (select* or order* or shop* or choice* or purchase*)).mp.
40	(choice$ or retail or spend or transaction*).mp.
41	35 or 26 or 37 or 38 or 39 or 40
42	Randomized controlled trial/
43	Controlled clinical trial/
44	Randomi?ed.ti,ab.
45	Randomly.ti,ab.
46	Trial.ti,ab.
47	Groups.ti,ab.
48	42 or 43 or 44 or 45 or 46 or 47
49	12 and 34 and 41 and 48
