# Supplementary material for: The Effectiveness of Interventions Delivered Using Digital Food Environments to Encourage Healthy Food Choices: A Systematic Review and Meta-Analysis
Source: Nutrients. 2021 Jun 30;13(7):2255. doi: 10.3390/nu13072255 (PMC8308236; doi:10.3390/nu13072255)
Supplement: Supplementary file 1 [file nutrients-13-02255-s001.zip › Nutrients Supp Files 29.06/Table S2 definition of healthy food and beverages.pdf]

Table S2: Defining 'healthy' and 'unhealthy' food and beverages

	Healthy	Unhealthy
**Macronutrients**	Less energy	More energy
	Less total fat	More total fat
	Less saturated fat	More saturated fat
	Less cholesterol	More cholesterol
	Less added sugar	More added sugar
	More fibre	Less fibre
**Micronutrients**	Less sodium	More sodium
**Food groups**	More wholegrains/cereals	Less wholegrains/cereals
	More vegetables	Less vegetables
	More fruit	Less fruit
	More lean meat/ poultry/ fish/ nuts/ seeds and legumes/beans	Less lean meat/ poultry/ fish/ nuts/ seeds and legumes/beans
	More low fat dairy	Less low fat dairy
	More water	Less water
	Less sugar sweetened drinks	More sugar sweetened drinks
	Less cake, biscuits, salted chips/crackers, lollies, chocolate, ice-cream, takeaway and processed meat	More cake, biscuits, salted chips/crackers, lollies, chocolate, ice-cream, takeaway and processed meat
	Less processed food	More processed food
**Diet quality**	Higher diet quality/ nutrient scores	Lower diet quality/ nutrient scores

*Note: these definitions are based on National Healthy Eating Recommendations [1]; and other nutrition profiling criteria [2].

References

1. Australian Government, National Health and Medical Research Council, and Department of Health and Ageing, *Australian Guide to Healthy Eating*, Department of Health and Ageing, Editor. 2017, Australian Government Department of Health: Canberra, Australia.
2. Lobstein, T. and S. Davies, *Defining and labelling 'healthy' and 'unhealthy' food.* Public Health Nutrition, 2009. **12**(3): p. 331-340.
